# The deubiquitinating enzyme USP5 promotes pancreatic cancer via modulating cell cycle regulators

**DOI:** 10.18632/oncotarget.19882

**Published:** 2017-08-03

**Authors:** Brajesh P. Kaistha, Anja Krattenmacher, Johannes Fredebohm, Harald Schmidt, Diana Behrens, Miriam Widder, Thilo Hackert, Oliver Strobel, Jörg D. Hoheisel, Thomas M. Gress, Malte Buchholz

**Affiliations:** ^1^ Department of Medicine, Division of Gastroenterology, Endocrinology and Metabolism, Philipps-University Marburg, Marburg, Germany; ^2^ Division of Functional Genome Analysis, German Cancer Research Center (DKFZ), Heidelberg, Germany; ^3^ Experimantal Pharmacology and Oncology (EPO Berlin-Buch), Berlin, Germany; ^4^ Institute for Bioprocessing and Analytical Measurement Techniques (IBA-Heiligenstadt), Heilbad Heiligenstadt, Germany; ^5^ Department of Surgery, University Clinic Heidelberg, Heidelberg, Germany

**Keywords:** pancreatic cancer, shRNA barcode library screen, ubiquitin specific peptidase, deubiquitinases, molecular pathogenesis

## Abstract

Pancreatic ductal adenocarcinoma (PDAC) is one of the most lethal solid tumors. With an overall five-year survival rate remaining below 6%, there is an explicit need to search for new molecular targets for therapeutic interventions. We undertook a barcode labelled short-hairpin (shRNA) library screen in pancreatic cancer cells in order to identify novel genes promoting cancer survival and progression. Among the candidate genes identified in this screen was the deubiquitinase *USP5,* which subsequent gene expression analyses demonstrated to be significantly upregulated in primary human pancreatic cancer tissues. Using different knockdown approaches, we show that expression of *USP5* is essential for the proliferation and survival of pancreatic cancer cells, tested under different 2D and 3D cell culture conditions as well as in *in vivo* experiments. These growth inhibition effects upon knockdown of *USP5* are mediated primarily by the attenuation of G1/S phase transition in the cells, which is accompanied by accumulation of DNA damage, upregulation of p27, and increased apoptosis rates. Since *USP5* is overexpressed in cancer tissues, it can thus potentially serve as a new target for therapeutic interventions, especially given the fact that deubiquitinases are currently emerging as new class of attractive drug targets in cancer.

## INTRODUCTION

Pancreatic cancer remains a major challenge to the biomedical community. Pancreatic ductal adenocarcinoma (PDAC), the most frequent type of pancreatic cancer, is one of the most lethal malignancies. It is typically characterized by late detection when it usually has metastasized, rendering a majority of patients unfit for surgical resection [1, 2]. Additionally, the disease is very resistant to the presently available regimens of chemo- and/or radiotherapies [3, 4] and the rate of incidence as well as mortality is predicted to increase for this menacing disease in the coming decades [2, 5]. Despite significant efforts to improve diagnostic and therapeutic options for the patients, the 5-year survival rate for PDAC patients has not improved much, illustrating a pressing need to further unravel the molecular basis of the aggressive biology of this malignancy. Using a variety of different screening strategies, several research groups, including ours, have previously uncovered candidate genes with novel tumor-promoting functions in PDAC, some of which have the potential to be exploited as novel diagnostic and/or therapeutic targets [6–10]. In this study, we have employed an unbiased cell-based screening approach to identify additional, hitherto unknown candidate genes in PDAC.

The Ubiquitin-Proteasome-System (UPS) plays an important role in protein homoeostasis in eukaryotes. It is involved in virtually all aspects of cellular activity by influencing proteolytic and non-proteolytic events, protein-protein interactions, protein localization, etc. [11, 12]. Ubiquitination of a target protein is achieved in a series of sequential steps catalyzed by three different enzymes, namely an E1 activating enzyme, an E2 conjugating enzyme and an E3 ligase. Ubiquitination is usually reversible and the ubiquitin moieties are recycled and released back into the system. This is achieved by a subclass of isopeptidases referred to as deubiquitynating enzymes, or in short, DUBs [12–14]. The human genome encodes about 98 DUBs [15, 16] and they have been implicated in different cellular and metabolic processes and diseases including inflammation, lung and heart injury, Parkinson’s disease and cancer [17–21]. Altered expression/function enables DUBs to act as either oncogenes or tumor suppressors depending upon their activity and the context [12, 16, 22]. There is an increasing body of evidence suggesting that DUBs represent a new class of promising drug targets that can be targeted more specifically than the proteasome due to their distinct association with particular genetic or biochemical pathways, especially in diseases like cancer [16, 23]. Previously, an SB transposon system-based screen demonstrated that the deubiquitynating enzyme USP9X plays an important tumor-suppressive role in pancreatic cancer development [7]. Here we describe an opposing function of another deubiquitynating enzyme, USP5, which has a physiologic function in the disassembly of branched polyubiquitin chains. The *USP5* gene locus has been reported to be genomically amplified in up to 10% of primary human pancreatic ductal adenocarcinomas [24] (also refer to the cBioPortal (http://www.cbioportal.org)). Our own results demonstrate that *USP5* is overexpressed on the mRNA level in the majority of PDAC cases and plays a tumor-promoting role *in vitro* and *in vivo*.

## RESULTS

### shRNA Barcode library screen to identify new therapeutic targets in pancreatic cancer

**Figure 1:**
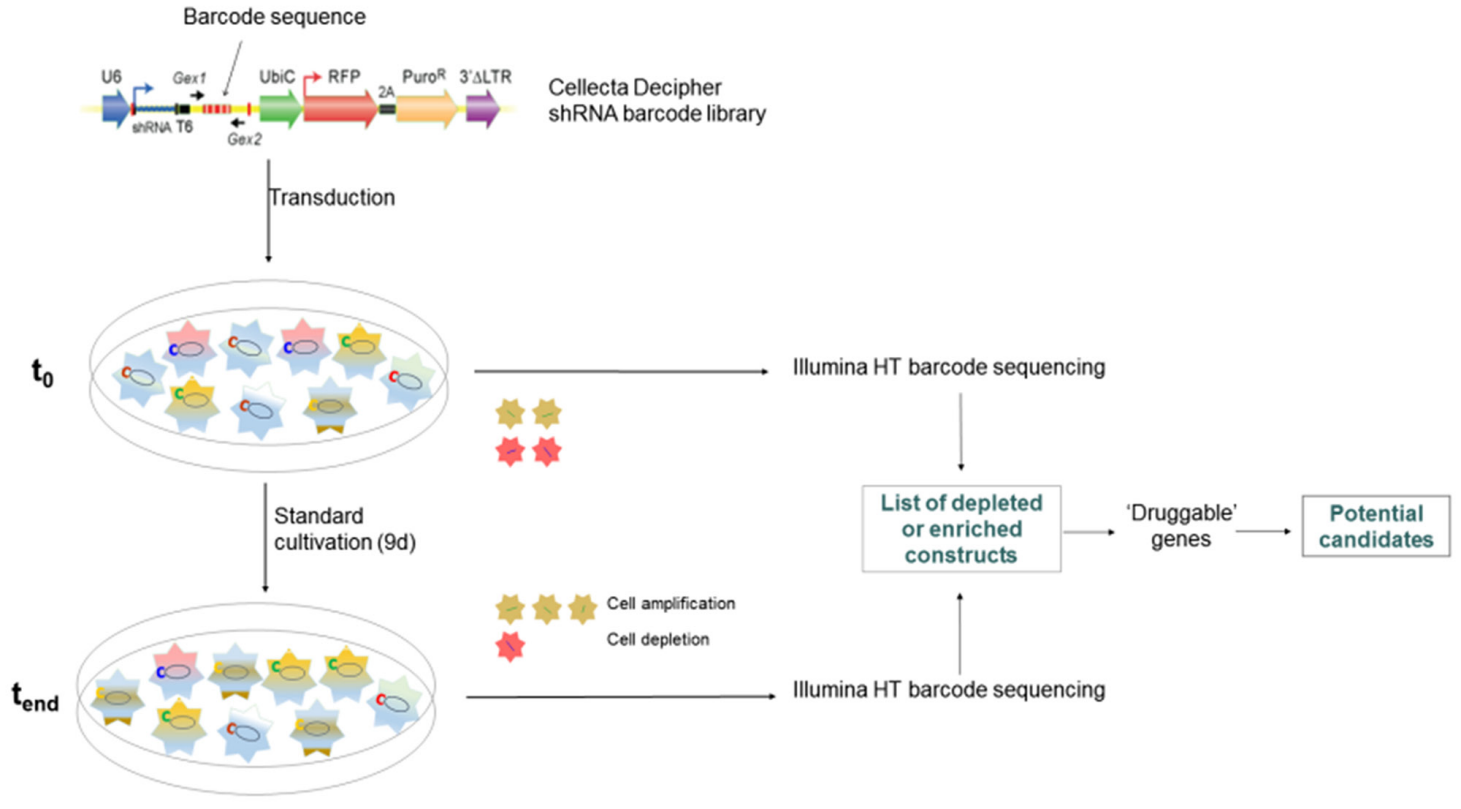
shRNA barcode library screening 10,000 genes were targeted by shRNA-mediated knockdown using modules 1 and 2 of the Cellecta Decipher library in BxPC3 pancreatic cancer cells in two separate screens carried out in identical manner. Genes were targeted by shRNA expression constructs, which are identifiable by barcode sequences. Cells were collected at t_0_ and t_end_, barcode sequences were amplified from genomic DNA and subjected to high-throughput sequencing and compared for their abundance.

To identify potentially new and hitherto uncharacterized genes in pancreatic cancer, we performed a barcode-labelled shRNA based screening (Figure 1) in BxPC3 pancreatic cancer cells using the Cellecta Decipher library. The relative abundance of individual barcodes (corresponding to specific shRNA expression constructs) was compared between the starting point and the endpoint of the experiment, termed “t_0_” and “t_end_” respectively, to identify shRNAs which were significantly enriched or depleted over the cultivation period of nine days (see Supplementary Materials for complete results). We then chose to focus on the genes for which the shRNA barcodes were depleted, as this can be interpreted as evidence for essential functions of the target gene in cell survival and/or proliferation (expression of the shRNA and thus downregulation of the target gene eliminates the recipient cell from the population). This gene list was then further pruned to only contain potentially druggable genes. The putative new druggable candidates identified are summarized in Table 1.

**Table 1:** List of putative druggable target genes

Cellecta ID	Gene symbol	Gene ID
CLL-H-049707	SMC2	10592
CLL-H-049708		
CLL-H-049709		
CLL-H-049711		
CLL-H-049710		
CLL-H-035753	GSPT1	2935
CLL-H-035754		
CLL-H-035752		
CLL-H-035755		
CLL-H-053393	USP5	8078
CLL-H-053390		
CLL-H-053392		
CLL-H-053391		
CLL-H-044118	PFAS	5198
CLL-H-044115		
CLL-H-044116		
CLL-H-046473	RAE1	8480
CLL-H-046475		
CLL-H-046471		
CLL-H-052434	TRIP13	9319
CLL-H-052436		
CLL-H-052433		
CLL-H-030361	CCT2	10576
CLL-H-030365		
CLL-H-030364		
CLL-H-028010	AHCYL1	10768
CLL-H-028007		
CLL-H-028006		
CLL-H-045957	PTPN23	25930
CLL-H-045955		
CLL-H-045959		
CLL-H-032831	DTL	51514
CLL-H-032830		
CLL-H-032827		
CLL-H-028474	ARHGAP10	79658
CLL-H-028473		
CLL-H-028475		
CLL-H-050482	STK3	6788
CLL-H-050486		
CLL-H-050485		
CLL-H-052752	TTK	7272
CLL-H-052750		
CLL-H-052751		
CLL-H-048171	SEPHS2	22928
CLL-H-048172		
CLL-H-048175		
CLL-H-053224	UPF2	26019
CLL-H-053225		
CLL-H-053227		

Shown are the Cellecta Decipher library-specific IDs of the barcoded shRNAs, the gene symbols of the candidate genes, and the corresponding NCBI gene IDs. Only target genes for which at least three independent shRNA constructs were consistently depleted from the population were considered for follow-up.

### *USP5* is up-regulated in pancreatic cancer tissue and promotes cell viability and proliferation

**Figure 2:**
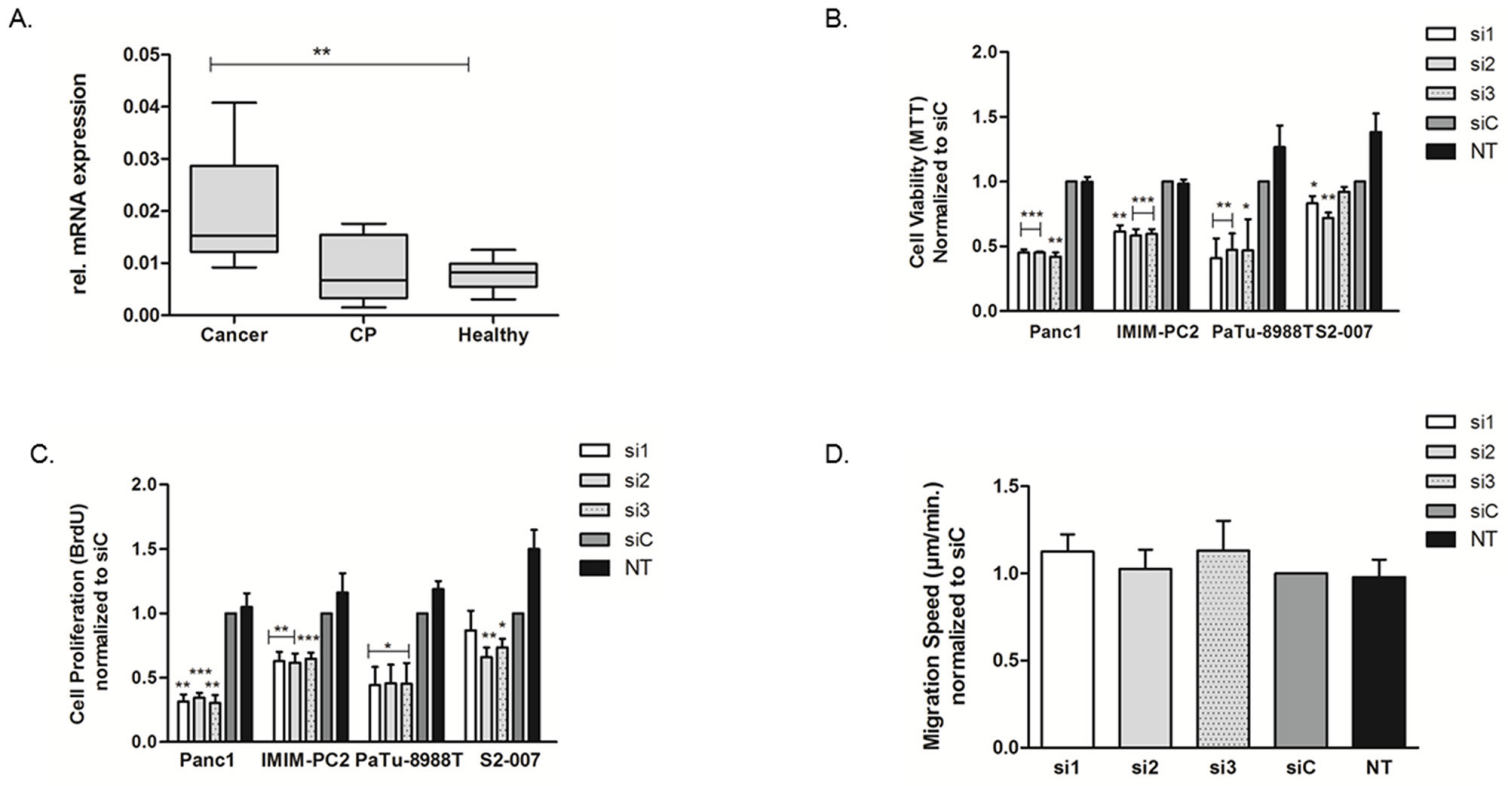
Upregulation of *USP5* and its role in in pancreatic cancer *USP5* mRNA expression was quantified by realtime PCR (qRT-PCR) and normalized to ribosomal protein, large, P0 (RPLP0) mRNA levels. **(A)** Expression in malignant tissues (n=10) was significantly increased compared to healthy controls (n=8) or chronic pancreatitis tissues (n=6). CP = Chronic Pancreatitis. **(B, C)** Transient loss of *USP5* function significantly reduced proliferation and viability of four different pancreatic cancer cell lines as assessed by their ability to metabolize MTT reagent (B, viability index) or incorporate BrdU agent (C, proliferation index) as compared to non-silencing control siRNA (siC) or untreated cells (NT). **(D)** *USP5* knockdown however had no impact on the migration speed of PaTu-8988T cells, as assessed by Time Lapse Microscopy and automated cell tracking. si1, si2 and si3 = three specific siRNAs against *USP5*. siC = non-silencing control. NT = untreated cells. *p<0.05, **p<0.01, ***p<0.001; Student’s T-Test. n≥3 for each experiment.

Among the novel candidate genes, *USP5* showed the highest level of overexpression in primary human PDAC as assessed by qRT-PCR. A modest increase in *USP5* mRNA expression was already evident in chronic pancreatic tissues as compared to the healthy tissues, while ductal adenocarcinoma tissues showed significant over-expression of *USP5* compared to both chronic pancreatitis as well as healthy pancreatic tissues (Figure 2A).

*USP5* mRNA was readily detectable in various cell lines tested. Eight different pancreatic cancer cell lines, including representatives of primaries (Panc-1, IMIM-PC2, BxPC-3 and MiaPaca-2 [25, 26]), and representatives of metastases (S2-007, S2-028, PaTu-8988T, [27, 28]) were analyzed. We did not observe any systematic differences in the expression levels of *USP5* among cancer cell lines of different origin or between the cancer cells and the non-transformed cell-line HEK293 (Supplementary Figure 1A).

To assess the functional relevance of this upregulation of *USP5*, we transiently silenced *USP5* gene expression using three different specific siRNAs in various pancreatic cancer cell lines. The downregulation of *USP5* protein was evident as early as 24 hours post siRNA transfection and remained stable for over 96 hours (Supplementary Figure 1B, upper panel).

Following transient silencing, viability of the cancer cells was measured using MTT assays, which showed that loss of *USP5* expression led to statistically significantly reduced viability of the cancer cells in all four cell lines tested (Figure 2B). These findings were further complemented by BrdU-ELISA based cell proliferation assays (Figure 2C) confirming compromised proliferative potential of pancreatic cancer cells in the absence of *USP5* expression. Interestingly, transient loss of *USP5* function in the non-transformed HEK293 cell line had almost no effect on cell viability except a modest inhibitory effect of siRNA2 (Supplementary Figure 2A). This suggests that dependency of cell survival and proliferation on *USP5* expression may be cancer cell specific.

We further analyzed the effects of *USP5* knockdown on the migratory potential of tumor cells using Time-Lapse microscopy and automated tracking of individual cell paths. Comparison of cells with and without knockdown, however, did not reveal any influence of *USP5* on the migration speed of the cells (Figure 2D).

### *USP5* knockdown leads to DNA damage, cell cycle arrest and apoptosis in PDAC cells

**Figure 3:**
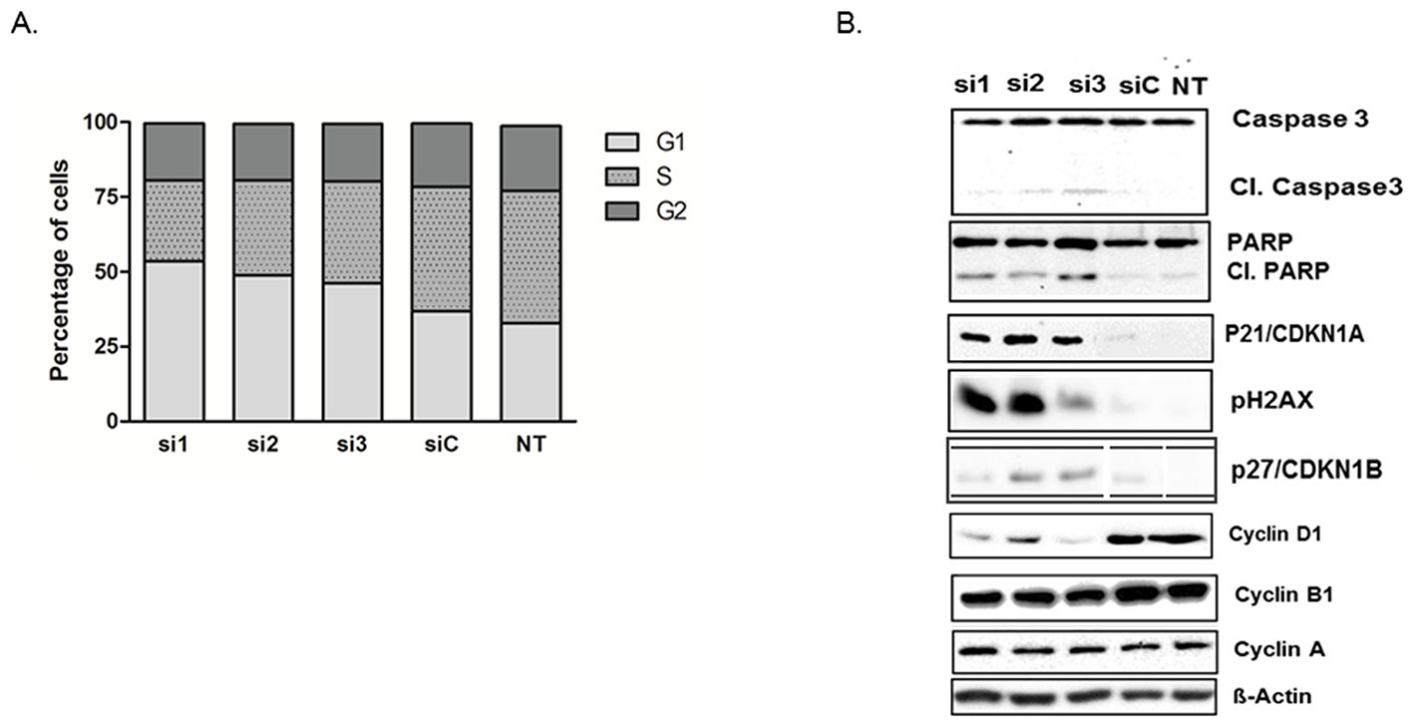
*USP5* knockdown leads to DNA damage, impaired cell cycle progression and apoptosis **(A)** Flow cytometry of transiently silenced PaTu-8988T cells revealed elevated proportions of cells lacking *USP5* function in the G1 phase and decreased proportions of cells in S-phase compared to control siRNA (siC) treated or untreated cells (NT). **(B)** Western blot analyses revealed increased PARP and Caspase 3 cleavage and elevated levels of phos. H2A.X, as well as increased levels of p21 and p27 and decreased levels of Cyclin D1 in the absence of *USP5* function. Other Cyclin proteins were unaffected. n≥3 for each experiment. si1, si2 and si3 = three specific siRNAs against *USP5*. siC = non-silencing control. NT = untreated cells.

Consistent with its role as a deubiquinating enzyme, *USP5* knockdown in pancreatic cancer cells led to accumulation of polyubiquitinated proteins (Ub2-4) (Supplementary Figure 2B). Cell cycle analyses of PaTu-8988T cells with *USP5* knockdown revealed significantly elevated proportions of cells in the G_1_-phase of the cell cycle compared to control siRNA treated or untreated cells, while the proportion of cells in S-phase were reduced accordingly. (Figure 3A). To further analyze the mechanisms mediating this cell cycle arrest, Western blot analyses were performed. *USP5* loss led to significant increase in p21 (CDKN1A) and p27 (CDKN1B) levels with simultaneous decrease in CyclinD1 in the cancer cells. In addition, there was clear evidence for accumulation of DNA damage, as evidenced by strongly increased levels of phos.H2A.X, as well as induction of apoptosis, as indicated by cleavage of both PARP and Caspase-3 (Figure 3B).

### *USP5* deficiency inhibits anchorage-independent cell growth and tumor formation *in vivo*

**Figure 4:**
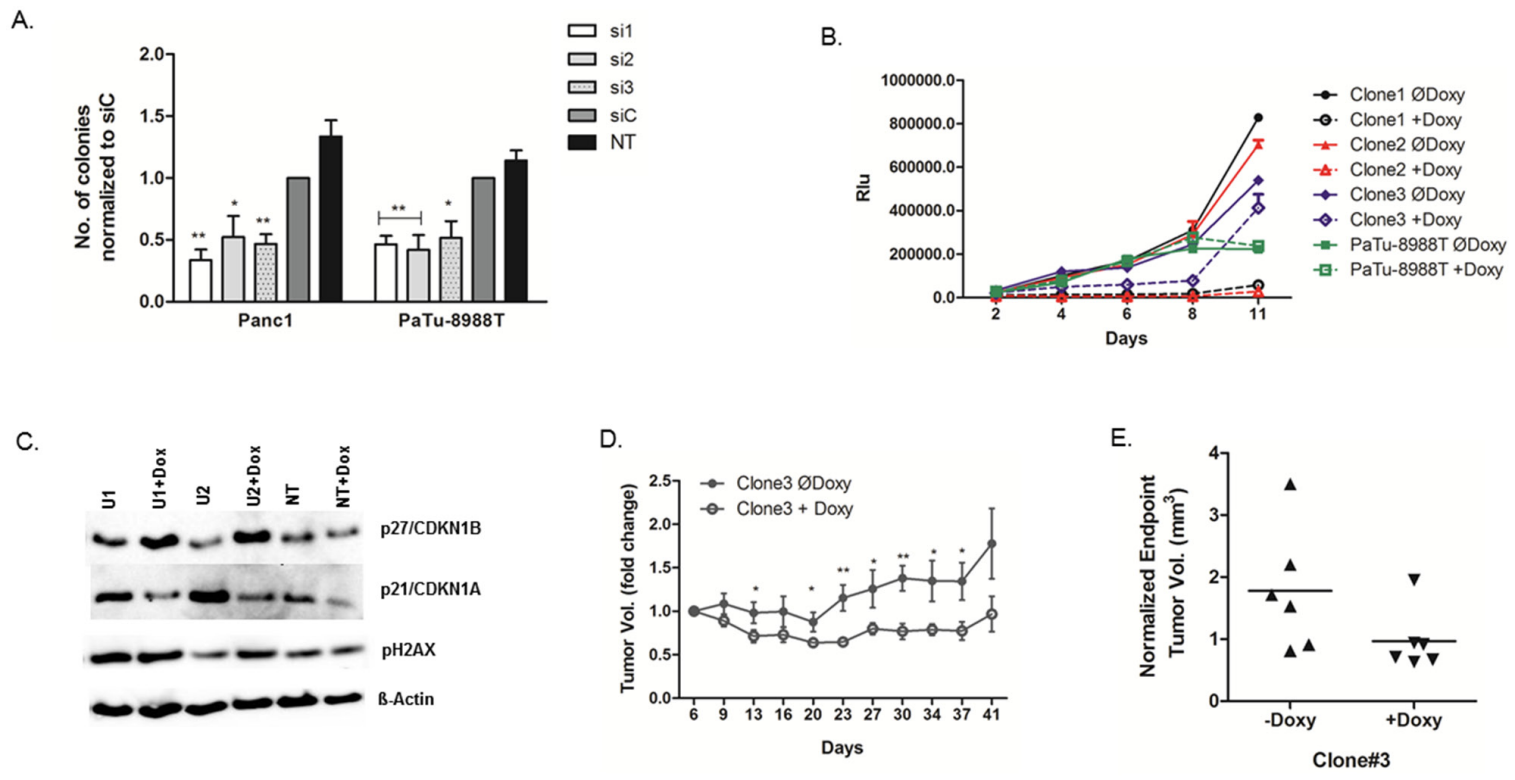
*USP5* is essential for transformation and tumor growth **(A)** Cells were transfected with *USP5*-specific siRNAs, non-silencing control siRNA, or left untreated, and subsequently grown under non-adherent conditions in soft agar. Cells lacking *USP5* expression formed significantly fewer colonies. **(B)** Lentiviral clones carrying three different doxycycline inducible shRNA constructs against *USP5* were generated from PaTu-8988T cells, grown under non-adherent conditions using the liquid overlay method, and cell viability assessed in regular intervals by chemiluminiscent assays. Induction of shRNA expression profoundly inhibited growth of the cells, while parental PaTu-8988T cells were unaffected by doxycycline treatment. **(C)** Western blot analyses of stably transfected clones revealed elevated levels of phos.H2A.X and p27 in the absence of *USP5* function, while p21 levels were decreased compared to non-induced cells. **(D)** For *in vivo* studies with shRNA clone #3, cells were xenografted subcutaneously into nude mice (n=6 per group). Doxycycline-induced knockdown of *USP5* significantly impaired tumor growth compared to control mice as assessed by caliper measurements. **(E)** Volumetric analyses of explanted tumors confirmed reduced tumor sizes in response to doxycycline-mediated *USP5* knockdown. si1, si2 and si3 = three specific siRNAs against *USP5*. siC = non-silencing control. NT = untreated cells. *p<0.05, **p<0.01.

We next investigated *USP5* function in clonogenic growth using two different 3D cell culture systems. Soft agar assays using transient RNAi revealed that Panc1 and PaTu-8988T cells lacking *USP5* expression formed significantly fewer colonies compared with control-treated cells (Figure 4A). To corroborate these findings, we generated lentiviral shRNA clones with doxycycline-inducible *USP5* knockdown in PaTu-8988T cells to study the impact of *USP5* loss over an extended time-period. Cells were cultured for eleven days using the liquid overlay technique in the presence or absence of doxycycline to induce *USP5* knockdown and cell viability repeatedly assessed by chemiluminescence assays. While parental PaTu-8988T cells were not influenced by the presence of doxycycline, all 3 individual clones were profoundly growth-inhibited (Figure 4B) upon induction of *USP5* knockdown with doxycycline (Supplementary Figure 1B, lower panel). Similar to the results from transient RNAi experiments, we also observed upregulation of p27 and increased levels of phos. H2A.X in Western blot analyses in response to doxycycline-induced *USP5* knockdown. Surprisingly though, in contrast to siRNA-mediated *USP5* knockdown, p21 levels decreased uniformly instead of increasing upon shRNA induction in the clones, indicating that p27 upregulation is the more relevant mediator of growth inhibition in this system (Figure 4C). In line with this observation, flow cytometric analyses of doxycycline-induced knockdown clones revealed elevated levels of cells in the G2 rather than the G1 phase of the cell cycle compared with control cells (Supplementary Figure 2C).

In parallel, clone #3 was used to establish xenograft tumors *in vivo* by subcutaneously implanting cells mixed with matrigel in athymic nude mice. Doxycycline-induced knockdown of *USP5* expression significantly attenuated tumor growth compared to control animals, as evidenced by both, external monitoring of tumor growth (Figure 4D) as well as comparison of final volumes of explanted tumors (Figure 4E).

## DISCUSSION

Pancreatic cancer is the fourth leading cause of cancer-related deaths in the United States [29] and is projected to become the second leading cause by 2030 [30]. This rise in mortality is compounded further by the fact that the choice of therapeutic options available is still very limited for this malignancy, and tumors tend to rapidly acquire resistance to the cytotoxic drugs [31, 32] thereby leaving little options for treatment. In this context, we performed a barcoded short hairpin RNA screen and identified the deubiquinating enzyme *USP5* as a potential novel target gene in pancreatic cancer.

The USP family of proteins has already been implicated in a variety of tumor-associated roles, be it tumor promoting or inhibiting. In fact,*USP5* itself has been reported to have a tumor inhibiting role in different disease models. It is a known regulator and stabilizer of *p53* transcriptional activity [21]. In melanoma cells, *USP5* has been reported to modulate FAS expression in a p53 dependent manner, thereby controlling growth [34]. However, here we report a novel aspect of *USP5* function in cancer independent of *p53*. Using 2D and 3D *in vitro* experiments as well as *in vivo* models, we show that USP5 is important for pancreatic cancer growth. Since *p53* is either mutationally inactivated or deleted in the vast majority of PDAC cases (as well as all cell lines used in our experiments), our data adds another layer to the functionality of this protein.

*USP5* knockdown in pancreatic cancer cells leads to accumulation of polyubiquitinated proteins (Ub2-4), whereas monomeric ubiquitin (Ub) is diminished. This is consistent with the previous findings that inhibition of DUBs leads to buildup of Ub-polymerized proteins [33, 34]. These polyubiquitinated proteins may then hinder cell growth via different mechanisms in different model systems. *USP5* knockdown has been implicated in cell cycle regulation before. However, contrary to the melanoma model, where downregulation of *USP5* leads to cell-cycle arrest in the G2/M phase as reported by Potu et. al. [34], in pancreatic cancer, the cells were arrested in the G1/S phase. It can be speculated that this difference is due to the fact that melanoma cells have an intact p53 function which then controls the transition from G2/M phase in these cells [35, 36]. However, in the absence of intact *p53* function in pancreatic cancer cells, *USP5* still plays a critical role in cell cycle progression. Silencing of *USP5* leads to cell cycle block in G1/S phase and this is facilitated by upregulation of p27, and down regulation of Cyclin D1. Intriguingly, the cell cycle regulator p21 was also strongly and reproducibly upregulated in response to transient siRNA-mediated *USP5* knockdown, but this effect was reversed in stably transfected clones in which *USP5* downregulation was mediated by doxycycline-inducible shRNA constructs. Possibly this difference reflects a combined effect of the unspecific cellular stress of transfection and the parallel loss of *USP5* function in the transient siRNA experiments. Absence of p21 upregulation in the stably transfected clones, however, suggests that the specific cell cycle inhibitory effects of *USP5* downregulation are mainly mediated via p27.

Loss of *USP5* leads to accumulation of DNA damage in pancreatic cancer cells as evidenced by elevated levels of phos.H2A.X. Ubiquitin specific peptidases have previously been associated with DNA damage response pathways, either by directly influencing ubiquitination of histones or by regulating relevant pathways such as the Fanconi anemia pathway [37, 38]. USP5 itself has been implicated previously in DNA damage pathway [39] where it was shown to play a role in efficient Double Strand Break (DSB) repair via homologous recombination (HR) and delayed disappearance of phos.H2A.X foci. In fact, inhibition of UDP genes has also been explored for use in combination therapy with DNA damage inducing drugs [40, 41]. However, in our hands, concomitant treatment of cells with Mitomycin C (MMC) along with *USP5* knockdown did not significantly enhance DNA damage response and phos.H2A.X accumulation (data not shown).

Taken together, our data support a novel important role for *USP5* in maintenance of chromosomal integrity in pancreatic cancer. Our results are consistent with the hypothesis that loss of *USP5* leads to accumulation of DNA damage, which in turn leads to accumulation of p27, cell cycle pertubation, and increased apoptosis rates. These data thus add to a growing body of evidence suggesting central (and partly context-dependent) roles of USP family proteins in tumor-relevant cellular processes. Indeed, USP family genes have been proposed as biomarkers in different malignancies [21, 42–45], and further research will have to show if USP5 may be suitable as a novel diagnostic marker and/or novel therapeutic target in pancreatic cancer.

## MATERIALS AND METHODS

### Barcode labelled shRNA based dropout screen

The screening method is described in detail in the supplementary material. In brief, shRNA-mediated knockdown of 10,000 genes was performed using modules 1 and 2 of the Decipher library (Cellecta, Mountain View, CA, USA) in two screens under identical conditions. The majority of genes were targeted by two or more individual shRNA expression constructs (average: five), which are identifiable by barcode sequences. BxPC-3 cells were infected with lentiviral particles containing either module 1 or 2 at a multiplicity of infection of below 0.3. Positively infected cells were selected by addition of 0.5 μg/ml puromycin 24 hours after infection. Selection took place for 48 hours, after which puromycin was removed and cells were allowed to recover for another 48 hours. A sample was taken for the *t*_0_ reference time point and then cells were cultured for additional 144 hours. This was the t_end_ reference time point. Barcode sequences were amplified from genomic DNA and subjected to high-throughput sequencing (detailed methodology in Suppl. Material) and then compared for enrichment or depletion. Only target genes for which at least three independent shRNA constructs were consistently depleted from the population were considered for follow-up.

### Cell lines and primary tissues

The human pancreatic adenocarcinoma cell lines Panc1, PaTu-8988T, BxPC3 and IMIMPC2 were used in this study. Panc1 and BxPC3cells were obtained from the German Collection of Microorganisms and Cell Cultures (DSMZ, Germany). PaTu-8988T cells were kindly provided by H. P. Elsässer (Cytobiology and Cytopathology Institute, Philipps University, Marburg, Germany). IMIM-PC2 cells were kindly provided by F.X. Real (CNIO, Madrid, Spain). These cancer cell lines were maintained in Dulbecco’s modified minimal essential medium (GIBCO, USA) supplemented with 10% FCS (GIBCO, USA) and cultured at 37°C/ 5% CO_2_.

Cancer cell line identities were verified using the GenomeLab Human STR Primer Set (Beckman Coulter, Krefeld, Germany) on a CEQ8800 sequencer (Beckman Coulter) according to the manufacturer’s protocol. STR data were submitted to on-line verification tool of DSMZ (www.dsmz.de) to confirm identity of human cell lines.

Surgically resected PDAC and chronic pancreatitis tissues samples were procured from the surgery department of the University of Heidelberg. Samples of normal pancreatic tissue were obtained from healthy donors. Informed consent in writing was obtained from all patients prior to using tissue samples. The study was approved by the ethics committee at the University of Heidelberg, Germany.

### siRNA and lentiviral transfections

PaTu-8988T, Panc1, S2-007 and IMIMPC2 cells were transfected with siRNAs using siLentFect Lipid Reagent (Bio-Rad, Germany) according to the manufacturer’s protocol. Unless stated otherwise, transient transfection was always done with 1x10^5^ cells per well in a 6-well plate format. Three specific siRNAs targeting *USP5* gene were used: Hs_*USP5 si1*, Hs_*USP5 si2;* Hs*_USP5 si3* (Ambion, Life Technologies, Germany); henceforth only distinguished as si1, si2, si3 respectively. Silencer® Negative Control #2 siRNA (Ambion, USA) was used as a non-silencing control (siC). Untreated cells, designated NT, were used as additional control.

For inducible repression of *USP5*,*USP5*-specific shRNA constructs were cloned into the tetracycline-inducible pLKO-U6-Tetr-on vector backbone (kindly provided by Dr. Stephan Hahn, Dept. of Gastroenterology, University of Bochum). For oligonucleotide sequences see Suppl. Material. Plasmid DNA was extracted and transfected into PaTu-8988T cells using jetPEI (PEQLAB GmbH, Germany) transfection reagent. Stably transfected cells clones were selected by puromycin (Sigma).

### RNA extraction, cDNA synthesis and qRT-PCR

RNA from cell lines was extracted using the peqGold Total RNA Kit (PEQLAB GmbH, Germany) according to the manufacturer’s protocol. For extraction of RNA from tissues, samples were homogenized in liquid nitrogen using a mortar and pestle. RNA was extracted using the RNeasy Mini Kit (Qiagen) following the manufacturer’s protocol.

1μg total RNA was used for first-strand cDNA synthesis using the Omniscript RT Kit (Qiagen) as per the manufacturer’s protocol. Quantitative real time Reverse Transcription PCR (qRT-PCR) was performed using SYBR Green MasterMix (Applied Biosystems, USA) on a 7500 Fast Realtime PCR system (Applied Biosystems). The complete list of primer pairs used for qRT–PCR is provided in the Supplementary Materials.

### Protein extraction and western-blotting

For protein extraction, cells were collected together with medium and centrifuged at 1600 rpm at 4°C for 5 min. Pellets were washed twice with ice-cold PBS and then re-suspended in 200μl lysis buffer [9, 10]. Protein concentration was assessed using Protein Assay Reagent (Thermo Scientific, USA). Western blotting was done by electrophoresing 10μg proteins on SDS–PAGE and subsequently transferring electrophoretically onto nitrocellulose membranes (Optitran, GE Healthcare Life Sciences, UK). Blocking was done in 5% non-fat dry milk in TBST for 2-4h at room temperature and then probed with appropriate primary and secondary antibodies. For details, please see Suppl. material.

### Cell viability and proliferation assays

Cell viability was measured by MTT assay as described before [9, 10]. Briefly, 2x10^4^ cells/well were seeded into 12-well plates and transfected the next day. After 72h of incubation, cells were incubated for 1.5h with MTT-reagent, solubilized and measured at 570 nm on plate reader. DNA synthesis as a direct measure of cell growth was measured using the Cell Proliferation ELISA, BrdU chemiluminescence Kit (Roche, Germany) according to the manufacturer’s protocol.

### 3D cell culture assays

Liquid overlay culture experiments were performed with low volume 384 well plates as described before [46] with one modification: plates were coated with 0.7% agarose (Seakem GTG Agarose, Lonza) in PBS and stored at 4°C until further usage. 500 cells were seeded in quadruplicate on these agarose-coated low volume 384-well plates (10μL per well, with or without 2μg/mL doxycycline). Cell viability was assessed on (day) d2, 4, 6, 8 and 11 days after seeding using CellTiterGlo®3D assay reagent (Promega) as per manufacturer’s specifications. To avoid evaporation effects, 2μL medium was supplemented to each well on d4 and d8 after seeding.

Soft agar clonogenic assays were performed in a 12-well plate format as described before [47]. Briefly, 6x10^3^ siRNA-transfected cells were re-seeded in a 0.33% DMEM-bacto-agar per well onto a bottom layer of 0.5% DMEM-bacto-agar. 1ml DMEM medium was added after the top agar had solidified. Medium was replaced every third day. Quantification was done by counting the number of colonies formed after 9 days of culturing.

### Time lapse microscopy

For evaluation of undirected cell migration, 35,000 cells were reseeded (36h after initial siRNA transfection) in collagen-coated 6-well tissue culture plates and placed under a microscope with temperature and CO_2_ control (Zeiss Cell Observer system, Carl Zeiss GmbH). Pictures of regions of interest were recorded in intervals of 10 min for a total time of 16-20h. The resulting time lapse video files were analyzed using the Time Lapse Analyzer software [48]. Cell paths were extracted for each individual cell and average velocities of migration calculated in μm/min.

### Flow cytometric analysis

48h after transfection with siRNAs, cells were trypsinized and centrifuged at 1,200 rpm for 3 min. After washing with PBS, pellets were thoroughly resuspended in 50-100 μl PBS and fixed with ice-cold ethanol (70%) and labelled with propidium-iodide mixture (details in[10]) and subsequently measured with the flow cytometer LSR-II (BD Biosciences, Heidelberg, Germany). For data evaluation, the software FlowJo ver. 7.6.5 (Tree Star Inc., Oregon, USA) was used.

### Mouse xenograft assay

2x10^6^ cells in 0.1ml 1:1 mix of PBS and matrigel were injected subcutaneously into the right flank of 6 week old female nude (NMRI-*Foxn1*^*nu*^/*Foxn1*^*nu*^) mice. shRNA expression was induced using doxycycline (Sigma Aldrich) in the drinking water (2mg/ml + 2% sucrose) while the control mice group received normal drinking water. Tumor sizes were measured twice a week using a caliper.

### Statistical analysis

Each experiment was repeated independently at least three times. Values are expressed as mean ± SEM of the triplicates, unless stated otherwise. Student’s *t*-test was used to analyze the difference between samples of interest vs. control. A *p* value of less than 0.05 was considered statistically significant.

## SUPPLEMENTARY MATERIALS FIGURES AND TABLE




