# Supplementary material for: The deubiquitinating enzyme USP5 promotes pancreatic cancer via modulating cell cycle regulators
Source: Oncotarget. 2017 Aug 3;8(39):66215–25. doi: 10.18632/oncotarget.19882 (PMC5630405; doi:10.18632/oncotarget.19882)
Supplement: Supplementary file 1 [file oncotarget-08-66215-s001.pdf]

The deubiquitinating enzyme USP5 promotes pancreatic cancer via modulating cell cycle regulators

SUPPLEMENTARY MATERIALS

1. Barcode labelled shRNA based dropout screen

Cell culture and Screening conditions

The pancreatic cancer cell line BxPC-3 was obtained from ATCC (Rockville, MD, USA). Cells were cultured in Roswell Park Memorial Institute (RPMI) 1640 medium (Life Technologies, Darmstadt) supplemented with 10% foetal calf serum (Life Technologies, Darmstadt) and 1% penicillin–streptomycin (Life Technologies, Darmstadt).

We targeted 10,000 genes for shRNA-mediated knockdown using modules 1 and 2 of the Decipher library (Cellecta, Mountain View, CA, USA) in two screens under identical conditions. The majority of genes were targeted by two or more individual shRNA expression constructs (average: five), which are identifiable by barcode sequences. The target cell line BxPC-3 was infected with lentiviral particles containing either module 1 or 2 at a multiplicity of infection of 0.3. Positively infected cells were selected by addition of 0.5 µg/ml puromycin 24 hours after infection. Selection took place for 48 hours, after which puromycin was removed and cells were allowed to recover for another 48 hours. A sample of 20 million cells was taken for the t_0 reference time point, shock-frozen and kept at −80°C. Cells were kept under selective pressure of 0.5 µg/ml puromycin, which was added after cells had attached to the substrate. After an additional 144 hours, cells were harvested, shock-frozen and kept at −80°C.

Barcode amplification

To determine the abundance of cells at t_0 and t_{end}, barcode sequences were amplified from genomic DNA and subjected to high-throughput sequencing. Amplification of barcode sequences was achieved by two rounds of PCR according to the following protocol. Cell pellets from samples obtained at the t_0 and t_{end} time points were thawed and subjected to ultrasonication at 50% intensity for 10 seconds on ice to shear genomic DNA into fragments of 1–10 kb. Genomic DNA was isolated from cell pellets using DNeasy Blood & Tissue Kit (69581, Qiagen, Hilden, Germany). For the first round of PCR, 50 µg of fragmented genomic DNA were used in eight 100 µl reactions containing: 1 µl Titantium Taq polymerase and corresponding PCR buffer (639210, Clontech of Takara Bio, Saint-Germain-en-Laye, France), 250 µM dNTP mix (M3015.4100, Genaxxon, Ulm, Germany), 3 µM HTS forward primer: 5′-TTCTCTGGC AAGCAAAAGACGGCATA-3′ and 3 µM reverse primer: 5′-TGCCATTTGTCTCGAGGTCGAGAA-3′ in nuclease-free water (AM9938, Life Technologies, Darmstadt, Germany). Desalted purity primers were obtained from Sigma Aldrich (Munich, Germany). Reaction mixtures were prepared on ice and subjected to a first round PCR starting with activation of the polymerase at 94°C for 3 minutes, followed by 16 cycles of denaturation at 94°C for 30 seconds, annealing at 65°C for 10 seconds, and elongation at 72°C for 20 seconds. Final extension was done at 68°C for 2 minutes. Second round PCR was done using 2 µl of the first-round reaction as template in three 100 µl reactions per condition. This time, primers GEX forward 5′-CAAGCAGAAGACGGCATACGAGA-3′ and GEX reverse 5′-AATGATACGGCGACCACCGAGA-3′ were used, while all other reagents remained the same. Thermal cycler settings were also unchanged, with the exception of a reduced elongation time of 10 seconds and a reduction of the cycle number to ten. Amplified barcode sequences were purified using PCR purification and gel extraction kits (28104 and 28704, Qiagen). Barcodes of module 1 and 2 were combined for each time point and quantified by next-generation sequencing (Genome Analyzer IIx, Illumina, San Diego, CA, USA). Each module contained a two-base-pair identifier attached to the barcode sequence for later discrimination.

Sequencing data analysis

Sequences were analysed to determine the abundance of each barcode using the software ‘Barcode Deconvoluter’ provided by Cellecta. The software counts the number of 18-nucleotide barcode sequences with a tolerance of two mismatches and assigns each barcode to the corresponding shRNA construct. Furthermore, each module was identified by two module-specific base pairs attached to the barcode sequence. Further analysis was done in Microsoft Excel. In order to compare reads of different modules and time points, each group of modules and time points was normalized to the average number of reads. Barcodes below 200 reads for t_0 were filtered out for reasons of quality assurance. Finally, drop-out ratios were calculated by dividing normalised reads of t_{end} by normalised reads of t_0 (expressed as % of t0).

Production of lentiviral particles

Lentiviral particles were produced using a second generation system described and engineered by Didier Trono’s group (Zufferey et al., 1997). HEK-293T cells were seeded in DMEM without antibiotics at a density

of 65,000 cells per cm^2 in a six-well format for single constructs or 175 cm^2 flasks for production of viral pools. Helper plasmids psPAX2 and pMD2.G were co-transfected with the construct carrier pRSI9 the following day using Lipofectamine (18324010, Life Technologies) and Plus Reagent (11514015, Life Technologies) according to the manufacturer's instructions. The next day, the medium was exchanged for high-serum DMEM (30% FCS). Supernatant containing lentiviral particles was harvested the following day, filtered through a 0.45 µm PES membrane (295–4545, Thermo Scientific) and stored in aliquots at −80°C.

2. *USP5*-specific shRNA lentiviral constructs

	5'→3'
pLKO – *USP5* siRNA#1(p-u1) sense / antisense	ccgggaggagaagtttgaattagacctcgaggtctaattcaaacttctcctcttttg<br>aattcaaaaagaggagaagtttgaattagacctcgaggtctaattcaaacttctcctc
pLKO - *USP5* siRNA#2(p-u2) sense / antisense	ccgggaggatgtgaagattgtcattctcgagaatgacaatcttcacatcctctttttg<br>aattcaaaaagaggatgtgaagattgtcattctcgagaatgacaatcttcacatcctc
pLKO - *USP5* siRNA#3(p-u3) sense / antisense	ccgggagggatgtgagggaaatgagctcgagcctatttccctcacatccctctttttg<br>aattcaaaaagagggatgtgagggaaatgagtccgagcctatttccctcacatccctc

3. List of primary antibodies used in Western blots

anti-Cyclin D1: Abcam, cat# ab16663

anti-Cyclin A: Santa Cruz, cat# SC-751

anti-Cyclin B1 (V152): Cell Signaling, cat# 4135

anti-p21: Cell Signaling, cat# 2947

anti-p27: Cell Signaling, cat# 2947

anti-phospho-H2A.X (Ser139): Cell Signaling, cat#

anti-PARP: Cell Signaling, cat# 9532

anti-Caspase-3: Cell Signaling, cat# 9664 & 9665

anti-Ubiquitin mAB P4D1: Enzo Life Sciences, cat# BML-PW0930

anti-Actin-HRP coupled: Sigma-Aldrich, cat# A3854

4. List of primer pairs used in the Quantitative real time Reverse Transcription PCR (qRT-PCR)

Ribosomal protein, large, P0 was used as the housekeeping control.

RPLP0_For: 5′-tgggcaagaacaccatgatg-3′; RPLP0_Rev: 5′-agtttctccagagctgggttgt-3′.

USP5_For: 5′-ccctgcaggccaagtcagt-3′; USP5_Rev: 5′-cagtctaagccgaaggtgaacttc-3′.

SMC2_For: 5′-acacttctgcaaccacagctttag-3′; SMC2_Rev: 5′-gttcccccctttctagtagcttttt-3′.

GSPT1_For: 5′-ctgcaaatgcaaacgtgctt-3′; GSPT1_Rev:5′-gagcaacgttagagaacaaatgctt-3′.

PFAS_For: 5′-tctcagcggactcgataatct-3′; PFAS_Rev:5′-tctgaaccacatgctgtttcct-3′.

TRIP13_For: 5′-tcacagcccatcgatttgagt-3′; TRIP13_Rev:5′-tttctgtctcttcctccagattttc-3′.

AHCYL1 _For: 5′-ctctgtgttccggccatga-3′; AHCYL1 _Rev:5′-tcctcttcaggccatccaaa-3′.

RAE _For: 5′-ctggccttgctgctcagatt-3′; RAE _Rev:5′-acccacttgggccttagga-3′.

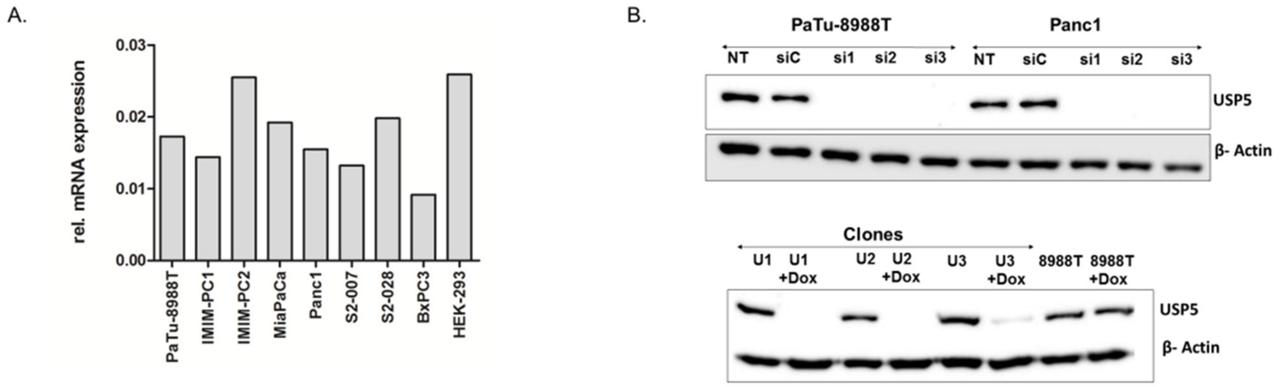

Supplementary Figure 1: **(A)** *USP5* expression in cell lines. *USP5* expression did not vary systematically between cell lines originating from primaries or metastases, or between transformed and non-transformed cells. mRNA expression was quantified by realtime PCR (qRT-PCR) and normalized to ribosomal protein, large, P0 (RPLP0) mRNA levels. **(B)** Effective knockdown of USP5 at protein level using transient siRNAs and the inducible clones. Western blot analyses using USP5 specific antibody showed the efficiency of knockdown at protein level. Upper panel shows knockdown in two different cell lines using transient siRNA based knockdown while lower panel shows the efficiency of doxycycline induction in three individual clones generated from PaTu-8988T cells. si1, si2 and si3 = three specific siRNAs against USP5. siC = non-silencing control. NT = untreated cells. U1, U2 and U3 = individual inducible USP5 clones; +Dox = Doxycycline.

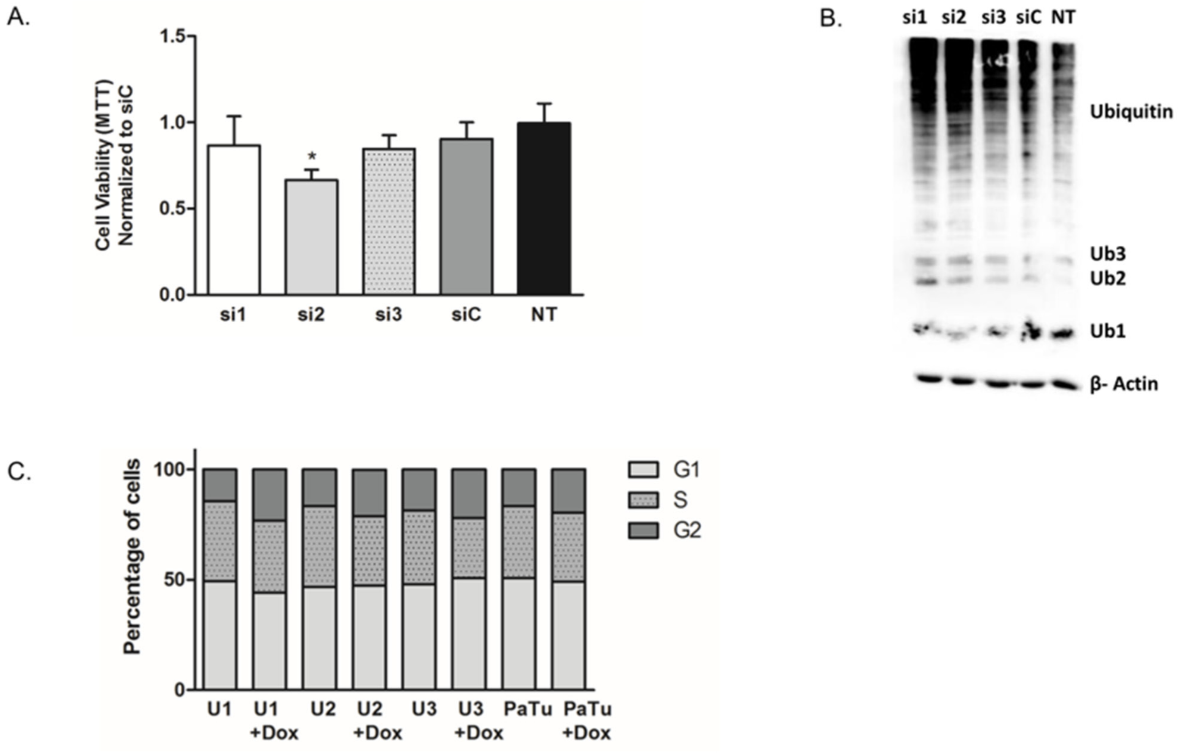

Supplementary Figure 2: **(A)** *USP5* knockdown does not affect the viability of HEK293 cells. Transient loss of *USP5* function did not systematically affect the viability of non-transformed HEK293 cells, as only si2 induced a moderate inhibition of the cells' ability to metabolize MTT reagent 72h after knockdown. si1, si2 and si3 = three specific siRNAs against USP5. siC = non-silencing control. NT = untreated cells. *$p<0.05$; Student's T-Test. **(B)** *USP5* knockdown leads to accumulation of polyubiquitinated proteins. Western blot analyses using a pan-ubiquitin antibody revealed that transient knockdown of *USP5* lead to accumulation of polyubiquitinated proteins and concomitant reduction of ubiquitin monomers in knockdown cells compared to controls. si1, si2 and si3 = three specific siRNAs against USP5. siC = non-silencing control. NT = untreated cells. **(C)** Flow cytometric analyses of inducible clones. Cell cycle analyses by flow cytometry revealed elevated proportions of cells in the G2-phase of the cell cycle in the clones generated from PaTu-8988T cells upon induction of USP5 knockdown with doxycycline compared to untreated cells. This was evident for all the three different clones tested, whereas doxycycline had no effect in the native PaTu-8988T cells. U1, U2 and U3 = individual inducible USP5 clones; +Dox = Doxycycline.

Supplementary Table 1: Complete results of the barcode screen.

See Supplementary File 1
